# Editorial: Inborn errors of immunity and mucosal immunity

**DOI:** 10.3389/fimmu.2023.1208798

**Published:** 2023-05-09

**Authors:** Elena Wen-Yuan Hsieh, Scott B. Snapper, Edwin F. de Zoeten

**Affiliations:** ^1^Department of Immunology and Microbiology, School of Medicine, University of Colorado Anschutz Medical Campus, Aurora, CO, United States; ^2^Section of Pediatric Allergy and Immunology, Department of Pediatrics, School of Medicine, University of Colorado Anschutz Medical Campus, Children’s Hospital Colorado, Aurora, CO, United States; ^3^Division of Gastroenterology, Hepatology and Nutrition, Boston Children’s Hospital and Harvard Medical School, Boston, MA, United States; ^4^Department of Pediatrics, Division of Gastroenterology, Hepatology and Nutrition, School of Medicine, University of Colorado Anschutz Medical Campus, Children’s Hospital Colorado, Aurora, CO, United States

**Keywords:** inborn errors immunity, inflammatory bowel diseases, innate immunity, inflammasome, anti-IL1 therapy

Inflammatory bowel disease (IBD) is a chronic inflammatory disease of the digestive tract which can present at any age, and is separated into two main subtypes, Crohn’s disease (CD) and ulcerative colitis (UC). While the two diagnoses demonstrate overlapping inflammatory pathology and clinical presentation, they have unique disease processes, evidenced both by disease characterization and known pathophysiology. Genome-wide association studies have identified an approximately 30% concordance of IBD-related genetic loci between CD and UC which suggests that the majority of susceptibility genes are unique to each subset (1). While treatment management strategies overlap in many cases between CD and UC, development of disease- and patient-specific precision medicine strategies is the ultimate goal. The discovery and study of inborn errors of immunity (IEI) elucidates mechanisms of human immunity and reveals candidate targets for personalized therapy. Inflammatory bowel disease (IBD) constitutes the primary presentation in up to 40% of IEI that present with auto/hyperinflammatory complications (2). Multiple etiologies contribute to intestinal pathology in monogenic IBD, including infections resulting from impaired antimicrobial immunity and disruptions in central and peripheral tolerance (3). The monogenic forms of IBD are rare, severe, and often refractory to conventional therapies (4, 5). Monogenic etiologies underlie 5-10% of very early onset (under 6 years old) IBD patients (VEO-IBD) (6–12). Monogenic IBD illustrates the importance and need for precision medicine in IBD care, as the underlying etiology dictates therapeutic choice and response. For example, cytotoxic T-lymphocyte associated protein-4 (CTLA-4) haploinsufficiency leads to defects in Tregs and a failed peripheral tolerance, with IBD as one of the manifestations of immune dysregulation (13). Abatacept, a fusion protein containing the extracellular domain of CTLA-4, has been successfully used as long-term therapy or a temporary therapy to quell the inflammation before hematopoietic stem cell transplantation (HSCT) (14–16). How different immunological defects impact intestinal epithelial cell composition, regulation, and function – and hence, intestinal homeostasis and health, is poorly understood. This knowledge gap constitutes a significant hurdle to guide current therapies and provide novel targets. This Research Topic gathers different contributions (research and review articles) describing how the study of different IEI has enlightened our understanding of mucosal immunity, and how such understanding can be translated into pathway-specific therapy in IBD.

In the mini-review by Ouahed, a selection of pertinent IEI resulting in monogenic IBD is described involving disorders in the intestinal epithelial barrier, phagocytosis, T and B cell defects, as well as those impairing central and peripheral tolerance. The contribution of disrupted gut-microbiota-host interactions in disturbing intestinal homeostasis among patients with intestinal disease is also addressed. The molecular mechanisms driving pathogenesis are reviewed along with the personalized therapeutic interventions, including HSCT, gene therapy, and small molecule inhibitors that target specific cellular/molecular pathways. Additionally, the growing multi-omic and interdisciplinary efforts in the study of monogenic IBD is also discussed.

Following the broad IEI and IBD review by Ouahed, Illig and Kotlarz focus their mini-review specifically on monogenic IBD etiologies that result from dysregulated inflammasome activity. Inflammasomes have been shown to play a central role in the defense strategy of intestinal epithelial cells (IEC), which is reflected by the expression of a diverse repertoire of inflammasome sensor proteins including NLR family CARD domain-containing protein 4 (NLRC4), pyrin-domain containing 3 and 6 (NLRP3, and NLRP6) (17). Previous studies have indicated that inflammasomes are implicated in IBD pathogenesis, as mucosal IL-1 production is significantly enhanced during active disease (18). Furthermore, higher IL-1 levels were detected in lipopolysaccharide (LPS)-stimulated peripheral blood mononuclear cells (PBMC) from CD patients and long-standing UC (19). Moreover, IL-1β signatures have been detected in macrophages/monocytes isolated from inflamed intestinal tissues of IBD patients by single-cell transcriptomics and deep immunoprofiling (20). Correspondingly, Liso et al. have recently demonstrated that failure to respond to anti-TNF therapy was associated with increased IL-1β in sera and colonic biopsy specimens from patients with UC (21). Taken together, these studies illustrate the importance of understanding the primary driver of the immunopathology in IBD to match specific therapies that target specific pathways that are dysregulated in specific patients, propelling precision medicine in IBD.

In line with the above mini-review, Shaul et al. demonstrated a positive clinical response in a single center retrospective study of patients with VEO-IBD with autoinflammatory phenotype (AIP) in the absence of monogenic disease treated with anti-IL1β (canakinumab) for >6 months. AIP was defined as confirmed IBD with associated signs of systemic inflammation in the absence of infection, including leukocytosis, markedly elevated inflammatory markers, and extraintestinal manifestations (recurrent fevers, oral ulcers, arthritis). Primary outcomes included clinical response in disease activity indices after 6 months of therapy. Secondary outcomes included rate of AIP signs and symptoms, growth, surgery, steroid use, hospitalizations, and adverse events. This study emphasizes the importance of a precision medicine approach in children with VEO-IBD, to maximize efficacy and minimize toxicity.

The study of genetic contributions to IBD, either as polymorphisms that lead to increased risk or causative genes in monogenic IBD, have served to enlighten our understanding regarding the regulation of such genes. For example, the gene encoding Nod2 is the first susceptibility gene that has been identified for CD (22, 23). However, mutations in *NOD2* also underlie Blau Syndrome (BS), an autosomal disorder characterized by granulomatous inflammation of the skin, joints, and eyes, but rarely the GI tract (24, 25). This difference is most likely due to the fact that the *CARD15* genetic abnormalities occurring in the two diseases differ with respect to the Card15 domain exhibiting mutational hits: the CD Card15 genetic abnormalities are present in the leucine-rich repeat (LRR) domain of Card15, the domain involved in NOD2 ligand engagement, whereas BS Card15 genetic abnormalities are present in the nucleotide-binding domain (NBD) of Card15, the domain mediating Nod2 oligomerization and interaction with downstream adaptors (26). The study of mutations in *NOD2* in Blau Syndrome by Mao et al. demonstrated the loss of Nod2 cross-regulatory function, which provides additional insight into its function of inflammatory disease. This study exemplifies how understanding the impact of different mutations within the same gene set the foundation to differentially immunomodulate in different patients who may otherwise be placed under the same umbrella treatment.

The study of inflammatory disorders due to IEI has shed light onto relative contribution, signaling cross-talks, specificity, and redundancy of each of those components in the orchestration of the immune response. Rodari et al., illustrate this concept nicely in their review of TGF-β signalopathies. Human TGF-β1 deficiency results in VEO-IBD, demonstrating the non-redundant role of TGF-β1 in suppressing intestinal inflammation and supporting the hypothesis that the aberrant TGF-β1/SMAD signaling observed in active lesions of IBD patients participate in disease pathogenesis (27). Importantly, targeting Smad7 by specific antisense oligonucleotides has been assessed, with mixed results, for the treatment of IBD (28–31).

Taken together, this Research Topic illustrates the diverse types/categories of IEI that underlie monogenic IBD. This research exemplifies how these genetic defects alter immune function at the mucosal site and provide novel insight into these immunological pathways, and their potential as novel targets for precision medicine in IBD. We hope that the reader will find this Research Topic a useful reference for the connection between IEI and mucosal immunity, and the utility of such line of research to help optimize therapy selection for IBD patients.

## Author contributions

EWYH, EFdZ, and SBC conceived this Research Topic together, edited the articles, and co-wrote the editorial. All authors contributed to the article and approved the submitted version.
